# Premature recruitment of oocyte pool and increased mTOR activity in Fmr1 knockout mice and reversal of phenotype with rapamycin

**DOI:** 10.1038/s41598-017-18598-y

**Published:** 2018-01-12

**Authors:** E. Mok-Lin, M. Ascano, A. Serganov, Z. Rosenwaks, T. Tuschl, Z. Williams

**Affiliations:** 1 Department of Obstetrics, Gynecology and Reproductive Sciences, University of California, San Francisco, USA; 2 Department of Biochemistry, Vanderbilt University, Nashville, USA; 3 Laboratory of RNA Molecular Biology, The Rockefeller University, New York, USA; 4 Center for Reproductive Medicine, Weill Cornell Medical College, New York, USA; 5 Department of Obstetrics and Gynecology, Columbia University Medical Center, New York, USA

## Abstract

While mutations in the fragile X mental retardation-1 (FMR1) gene are associated with varying reproductive outcomes in females, the effects of a complete lack of FMR1 expression are not known. Here, we studied the ovarian and reproductive phenotypes in an Fmr1 knockout (KO) mouse model and the role of mammalian target of rapamycin (mTOR) signaling. Breeding, histologic and mTOR signaling data were obtained at multiple time points in KO and wild type (WT) mice fed a control or rapamycin (mTOR inhibitor) diet. KO mice showed an earlier decline in ovarian reserve than WT mice with an increased proportion of activated follicles. mTOR and phosphorylated S6 kinase (p-S6K) levels, a measure of downstream mTOR signaling, were elevated in the KO ovaries. Rapamycin blocked these effects in KO mice, and increased the primordial follicle pool and age of last litter in WT mice. Our data demonstrates an early decline in reproductive capacity in Fmr1 KO mice and proposes that premature recruitment of the primordial pool via altered mTOR signaling may be the mechanism. Reversal of phenotypes and protein levels in rapamycin-treated KO mice, as well as increased reproductive lifespan of rapamycin-fed WT mice, suggest the mTOR pathway as a potential therapeutic target.

## Introduction

Abnormalities in the fragile X mental retardation-1 (FMR1) gene located on Xq27.3 are associated with a wide array of phenotypic, neurologic and reproductive conditions^1^. *FMR1* encodes for fragile X mental retardation protein (FMRP), an RNA-binding and translation-inhibiting protein highly expressed in the brain, oocytes and granulosa cells^2–4^. The 5′ promoter region of *FMR1* normally contains 5–44 cytosine-guanine-guanine (CGG) trinucleotide repeats. An expansion to >200 CGG trinucleotide repeats, termed a “full mutation”, results in DNA hypermethylation of the promoter region and silencing of FMR1, leading to a deficiency in FMRP^5,6^ and causing Fragile X syndrome (FXS), the most common inherited cause of mental retardation and a monogenetic cause of autism^1^. Expansion to 55–200 CGG trinucleotide repeats, termed a “premutation”, results in hypomethylation of the promoter region and elevated FMR1 mRNA levels^7,8^ causing fragile X-associated tremor/ataxia in males and fragile X-associated primary ovarian insufficiency (FXPOI) in females. Premutation in the FMR1 gene is the most common genetic cause of POI, the loss of ovarian function before age 40, accounting for up to 13% of familial cases^5^. Interestingly, while premutation carriers have been shown to have up to a 20-fold higher risk of developing POI than non-premutation carriers, women who are full mutation carriers – those who have a single copy of the full mutation – have not been found to be at increased risk^9^. However, the reproductive phenotype of a female homozygous for the FMR1 full mutation is not known. The aim of our study was to examine the effects of complete silencing of the FMR1 gene on ovarian reserve and reproductive capacity using a Fmr1 knockout (KO) mouse model.

We recently demonstrated that mTOR, a protein kinase in the phosphatidylinositol 3-kinase (PI3K) pathway, is a direct target of FMRP and that tissue from male FXS patients and Fmr1 KO mice expressed higher levels of mTOR^10^. mTOR is a key regulator of primordial follicle recruitment, balancing the suppressive effects of phosphatase and tensin homolog (PTEN); PTEN KO mice resulted in premature activation of the follicular pool only in the setting of active mTOR^11^.

We therefore hypothesized that the loss of functional FMRP results in decreased mTOR suppression, thereby accelerating recruitment and subsequent depletion of primordial follicles. To explore the disruption in the mTOR signaling pathway in Fmr1 KO mice, we designed a series of longitudinal experiments to assess differences in fertility, ovarian size, follicle counts and mTOR levels. We also assessed whether any observed effects in the KO mice could be reversed by administering rapamycin, an mTOR antagonist^12^.

## Materials and Methods

### Animals

FVB;129P-Fmr1^tm1Cgr^/J mice generated via homologous recombination using an exon-disrupted targeted vector with nil FMR1 RNA or protein expression were used for breeding, histology and protein analysis experiments (n = 84). The strain was maintained by breeding homozygous females with hemizygous males. Mice of the same strain were used as controls. All mice were maintained in a temperature- and light-controlled environment with a 12/12 h light/dark cycle and were treated in accordance with the principles and procedures of the National Institutes of Health Guide for the Care and Use of Laboratory Animals. Protocols were approved by the Institutional Animal Care and Use Committee of The Rockefeller University. All experiments were performed in accordance with relevant guidelines and regulations.

### Diet preparation

14 mg/kg of rapamycin (LC Laboratories, Woburn, MA) was microencapsulated by Southwest Research Institute (San Antonio, TX) with enteric coating material Eudragit S100 (Röhm Pharma, Germany) to bypass gastric breakdown and allow for effective drug delivery^13^. Encapsulated rapamycin was then incorporated into 5LG6 mouse chow (PMI Nutrition International, Brentwood, MO). Control mice received the same base diet with enteric coating without rapamycin.

### Ovarian size, protein and follicular analyses

KO and wild type (WT) female pups were euthanized at 3 weeks of age for follicular and protein analysis (n = 4). An additional 40 female pups were weaned and started on rapamycin or control diet for analysis at 6, 9, 12, 18 and 40 weeks of age (n = 8 per time-point). Gross weights of female mice were obtained prior to euthanization by cervical dislocation. Dissection of the ovaries was performed using a stereomicroscope at 3x magnification. Ovaries were washed in PBS and weighed on an analytical balance to obtain total ovarian weights. The right ovaries were immersed in liquid nitrogen (LN_2_, −196 °C) for protein extraction. A series of immunoblots using lysates prepared from these ovaries were performed to analyze mTOR levels in the WT and KO mice on control or rapamycin diet. As mTOR signaling leads to phosphorylation and activation of ribosomal protein S6 kinase (S6K;^14^), S6K and p-S6K levels were also measured as indicators of mTOR activity. Poly(A)-binding protein (PABP) was used as a loading control.

The left ovaries were fixed in 4% paraformaldehyde at 4 °C for 24 hours and embedded in paraffin for analysis of follicle numbers. Serial 5 µm sections were cut on a rotary microtome and stained with hematoxylin and eosin. Primordial, primary, secondary and antral follicles were counted on every tenth section based on a modification of previously established follicle counting methods^15,16^. Total number of follicles per ovary was estimated by multiplying the number of follicles on counted sections by 50 to account for every 10^th^ section and section thickness. A single researcher, blinded to the age, genotype and diet type of the mouse from which the section was obtained, conducted all follicle-counting experiments.

### Breeding experiment

KO and WT female mice were weaned at 21 days of age and started on the encapsulated rapamycin diet or control diet (n = 40). WT and KO female mice on the control diet were bred with WT males beginning at 8 weeks of age. Female mice were weighed and exposed to 1 male every 3 days during each 9-day breeding cycle. Litter sizes were recorded and pups were euthanized. Each subsequent breeding cycle was initiated 3 weeks after the last litter. Female WT and KO mice on the rapamycin diet were not bred while on rapamycin until a decrease in fertility was seen in the corresponding mice on control diet. Rapamycin was discontinued and replaced with control diet 2 weeks prior to initiating breeding in these mice to allow for a washout period. All mice were bred until 92 weeks of age or mortality, whichever occurred first.

### Statistical analyses

Chi-squared tests were used to analyze the proportion of primordial versus activated (primary, secondary and antral) follicles in the mice. Cox-regression with pairwise comparisons was utilized to assess differences in age of last litter. P< 0.05 was deemed statistically significant.

### Data availability

The datasets generated during and/or analyzed during the current study are available from the corresponding author on reasonable request.

## Results

### Ovarian size

**Figure 1:**
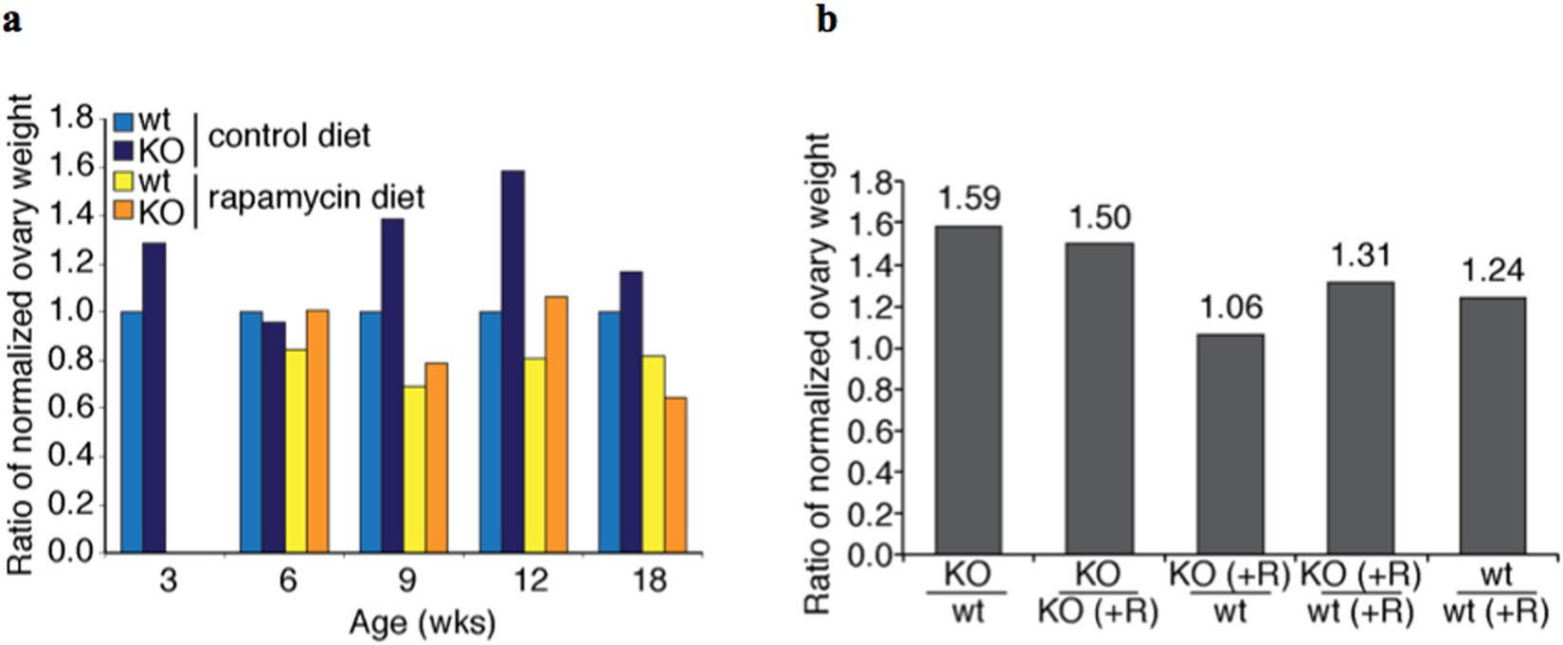
Effect of rapamycin on Fmr1−/− ovary size. Female mice were fed with control or rapamycin-containing diet, starting at the third postnatal week. Ovaries were harvested and weighed at indicated times, while also measuring the total mouse weight for normalization (n = 8, per time point). (**a**) Average ovarian weights, normalized to the WT control diet group are shown and colored based on genotype and diet. (**b**) To determine net mass difference at the twelve-week time-point, the ratios of the normalized ovarian weight for each of the genotype and dietary criteria indicated along the X-axis were calculated.

Ovaries from Fmr1 KO mice were heavier and larger than ovaries from age-matched WT controls, although this did not reach statistical significance (Fig. 1). The difference appeared greatest at 9 and 12 weeks of age, which may be related to an increase in pre-antral and antral follicles post-sexual maturity. Rapamycin reversed the difference in ovarian size, such that KO mice that received rapamycin developed ovaries of a similar size to that of WT controls. WT mice that received rapamycin also developed smaller ovaries compared to age-matched WT controls.

### Follicle counts

**Figure 2:**
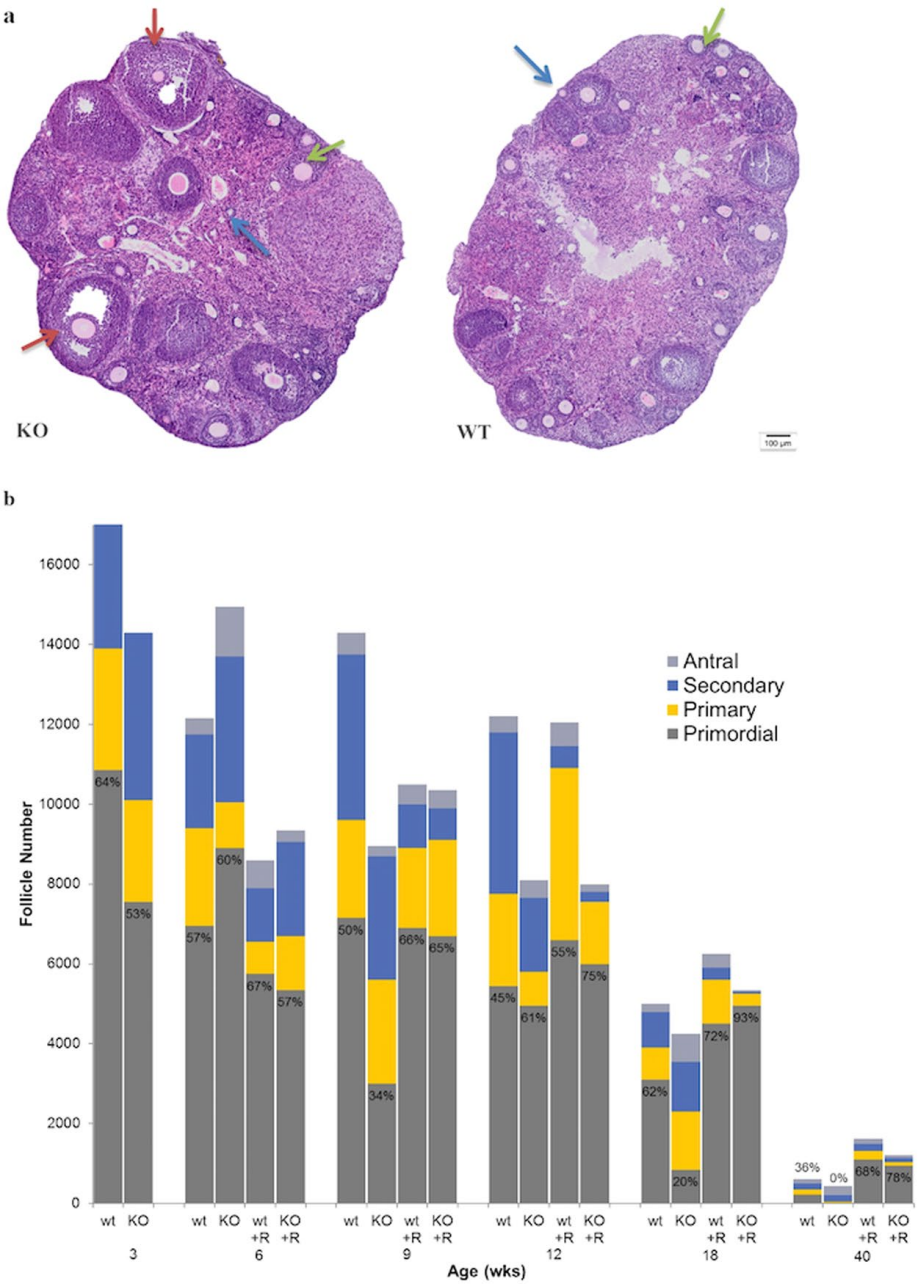
Differences in follicle numbers and distribution in Fmr1 KO ovaries. (**a**) H + E-stained ovarian sections from 9 week-old mice demonstrate the increased number of activated (primary, secondary and antral) follicles in KO compared to WT ovaries. Examples of primary follicles are indicated with blue arrows, secondary with green arrows and antral with red arrows. (**b**) Averaged follicle counts and proportion of primordial follicles among untreated and rapamycin-fed WT and KO mice are shown at indicated time-points (n = 8).

Total follicle count and number of primordial follicles were similar between the WT and KO mice at the early time-points (Fig. 2). At 18 weeks of age, within a mouse’s prime reproductive window, the WT and KO ovaries consisted of a similar total number of follicles; however, the WT mice had a significantly higher proportion of primordial follicles compared to KO (62 vs. 20%, p< 0.0001). While both groups had fewer than 1000 total follicles by 40 weeks of age, 36% of the remaining follicles in the WT ovaries were primordial whereas the KO mice had depleted their primordial pool (p< 0.0001).

Treatment of Fmr1 KO mice with rapamycin preserved primordial follicle counts. Fmr1 KO mice that received rapamycin had significantly more primordial follicles than their age-matched KO controls that did not receive rapamycin at 9, 12, 18 and 40 weeks of age (p< 0.0001), restoring them to levels that were at or above the WT controls. Treatment with rapamycin also significantly increased the primordial pool in WT mice at every time-point, such that 40 week-old Fmr1 WT mice receiving rapamycin had over 4-fold the number of primordial follicles than Fmr1 WT controls that did not receive rapamycin (1100 vs. 220, p< 0.0001).

### Protein levels

**Figure 3:**
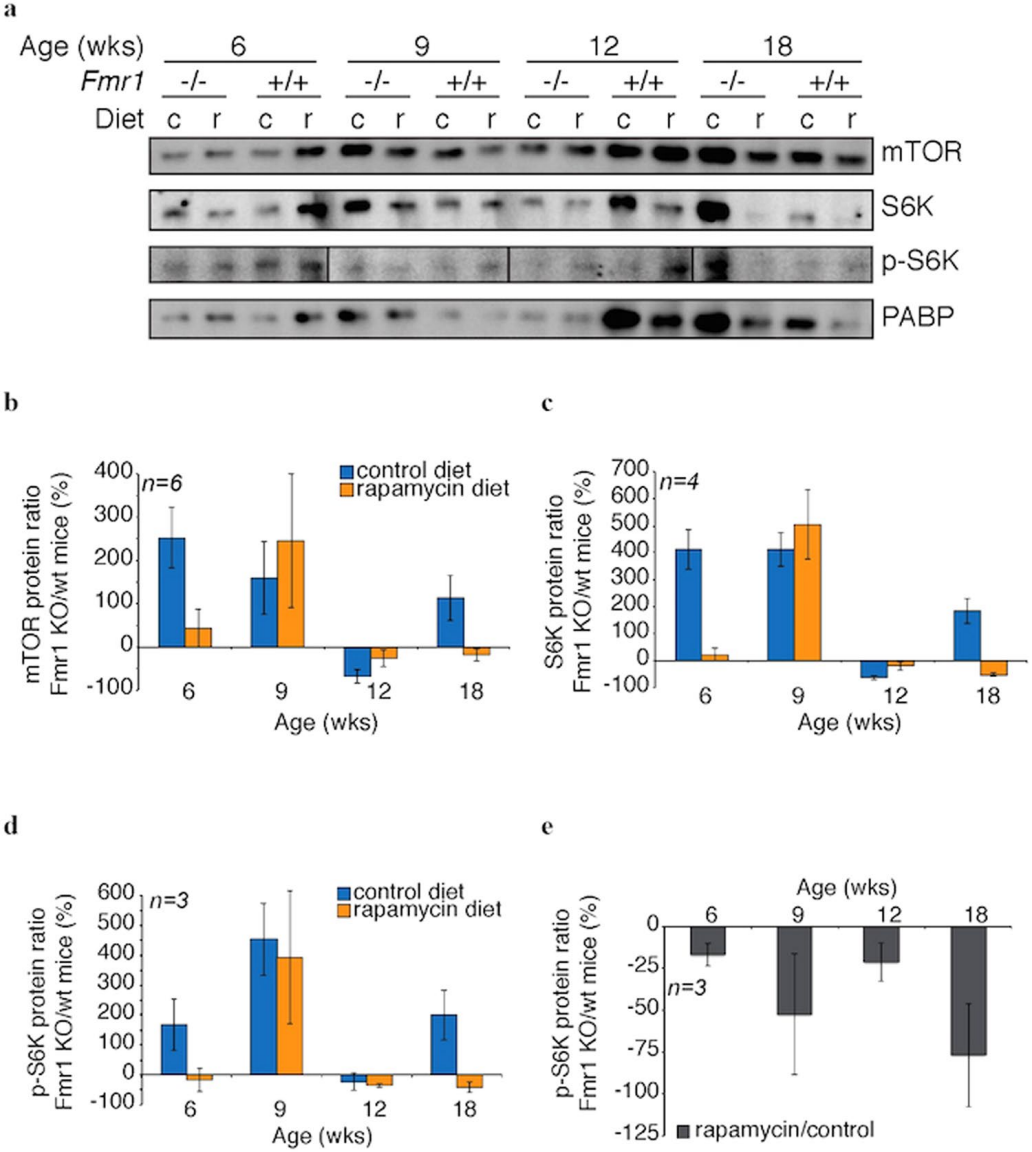
Effect of rapamycin on elevated protein levels of mTOR and S6K, two FMR1 targets. Lysates were prepared from ovaries of WT or Fmr1 KO mice, fed with control or rapamycin-containing diet, at indicated times. (**a**) Shown are representative immunoblots of mTOR, S6K, and phosphorylated S6K (p-S6K), using PABP as a loading control. (**b**) Quantitation of the immunoblot data (n = 6, SEM shown) of the ratio of mTOR protein in KO versus WT ovaries. (**c**) Quantitation of the immunoblot data (n = 4, SEM shown) of the ratio of S6K protein in KO versus WT ovaries. (**d**) Quantitation of the immunoblot data (n = 3, SEM shown) of the ratio of p-S6K protein in KO versus WT ovaries. (**e**) Treatment with rapamycin reduced the levels of p-S6K in KO mice (n = 3, SEM shown), compared to WT. A value of 0% would indicate no p-S6K change.

mTOR protein levels were elevated in Fmr1 KO ovaries compared to WT in 3 of the 4 time-points checked (Fig. 3). S6K levels were also elevated in KO mice, suggesting that S6K might be a post-transcriptional target of FMRP in ovaries. Eight FMR1 binding sites were previously identified within human S6K (RPS6KB1) transcript although its enrichment score in HEK293 cells only ranked it among the top 45% of targets^10^. The protein levels of mTOR or S6K did not correlate with rapamycin treatment. However, phosphorylated S6K levels were increased in three of the four time-points checked, indicating increased levels of activated S6K in KO mouse ovaries. Treatment with rapamycin reduced the levels of p-S6K (p< 0.05 at time-points 6 and 18 weeks), as would be anticipated if mTOR activity was responsible for its phosphorylation.

### Breeding rates

One WT mouse died at 21 weeks of age of unknown causes. No further mortalities occurred until 53 weeks of age, at which point additional mice began to experience age-related deaths. By 78 weeks, 26 of the 40 breeding mice participated in the breeding cycles (9 KO, 7 WT, 4 rapamycin-treated KO, 6 rapamycin-treated WT). At the completion of the experiment at 92 weeks, 2 Fmr1 KO, 2 WT, 0 rapamycin-treated KO and 6 rapamycin-treated WT mice remained.

**Figure 4:**
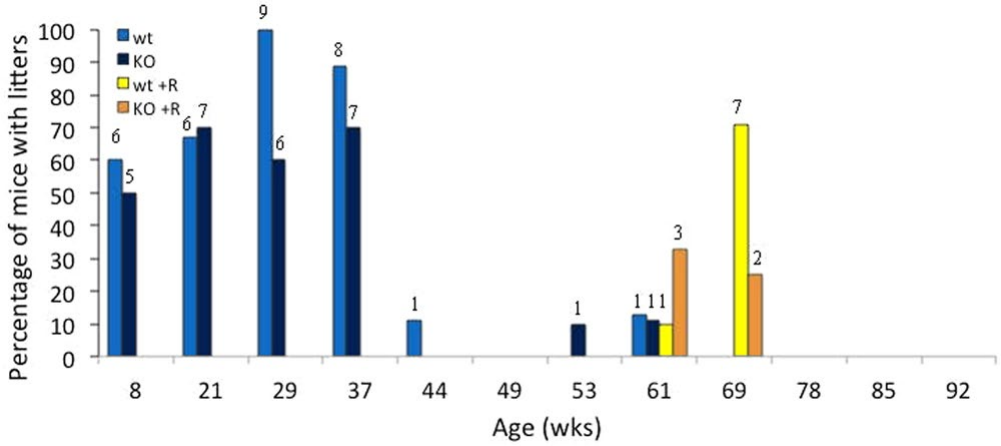
Effect of rapamycin on breeding rates. The percentage of mice that produced litters per breeding cycle are shown and colored based on genotype and diet (n = 40). WT and KO mice fed a control diet were bred at regular intervals beginning at 8 weeks of age. WT and KO mice on a rapamycin diet (WT + R, KO + R) were given a two-week washout period from rapamycin and placed on a control diet before initiating breeding. KO mice showed an earlier decline in breeding rates. Treatment with rapamycin extended fertility. Above each bar is the number of mice in each group for each time point.

Fmr1 KO and WT mice exhibited similar breeding rates from 8–21 weeks of age (Fig. 4). At 29 weeks of age, 60% (n = 10) of the KO group had litters compared to 100% (n = 9) of the WT mice. By 44 weeks, the WT group also showed a sharp decline in fertility - 1 (11%) WT mouse and 0 KO mice had a litter. While Fmr1 KO mice appeared to show an earlier decline in fertility than WT mice, this did not reach statistical significance (p = 0.116). Among WT mice, treatment with rapamycin increased the reproductive lifespan, such that WT mice on the control diet were nearly 4 times more likely to stop reproducing than rapamycin-treated mice at the same age (p = 0.009, HR 3.96, 95% CI [1.404, 11.167]). Rapamycin also prolonged fertility in Fmr1 KO mice, with a 2-fold increase in likelihood of having no litters compared to Fmr1 KO that received rapamycin, although this was not statistically significant (p = 0.137, HR 2.0223, 95% CI [0.799, 5.126]).

## Discussion

While it is known that women with a single copy of a full FMR1 mutation do not have impaired ovarian function, there are no reported cases of females with FMR1 full expansions on both alleles to date as that would require that the X chromosome inherited from the father have a full mutation, which has never been shown and may not be possible. As such, the effects of complete FMR1 silencing on reproductive capacity is not known. We sought to utilize the Fmr1 KO mouse model to study the effects of complete mRNA and protein deficiencies.

Although the Fmr1−/− strain has been available for two decades, this is the first longitudinal study to investigate the reproductive and ovarian phenotypes in these knockout mice. The original paper describing Fmr1 KO mice stated that the “female knockout mice were fertile, and the females had normal litter sizes” but did not assess ovarian phenotype or follow their long-term reproductive outcomes^17^. The results of our breeding experiment suggest that Fmr1 KO mice exhibit decreased fertility earlier than WT mice. The higher proportion of pre-antral and antral follicles and the corresponding increase in ovarian size of the KO ovaries show that the earlier decline in fertility may be due to an increased activation of primordial follicles resulting in diminished ovarian reserve, as opposed to a decrease in the initial primordial pool. Elevated mTOR and p-S6K levels in the Fmr1 KO ovaries indicate that the loss of FMRP suppression results in increased mTOR expression and activity; this change in mTOR signaling is likely the mechanism behind the observed reproductive and ovarian phenotypes in Fmr1 KO mice.

Consistent with this hypothesis were the observed decreases in p-S6K levels and reversal of phenotypes with rapamycin treatment. Ovaries from Fmr1 KO mice who received the mTOR inhibitor were smaller in size, similar to their age-matched WT controls, and had an increased proportion of primordial follicles. These histologic findings corresponded to a trend towards an increased reproductive lifespan, with 25% of the rapamycin-treated KO mice continuing to breed 8 weeks beyond the last KO mouse on control diet. Since the mouse estrus cycle is approximately 3 days, an additional 8 weeks of breeding in mice is similar to an extension of 18 months of fertility in women.

While rapamycin and the mTOR pathway have been extensively studied in oncology and neurobiology, this is also the first known study to describe the long-term reproductive effects of rapamycin in WT mice. Rapamycin (sirolimus) given as an immunosuppressant following kidney transplantation has been shown to cause reversible amenorrhea in female patients^18^. Our findings of a prolonged reproductive lifespan and increased primordial pool in rapamycin-treated WT mice suggest that these effects in humans may be related to a suppression of mTOR-stimulated activation of primordial follicles. Stopping rapamycin releases this inhibition and allows for follicular recruitment again, which is consistent with the resumption of menses seen in women following discontinuation of sirolimus. Our findings in the rapamycin-treated mice carry implications for further development of interventions targeting mTOR for age-related diminished ovarian reserve, primary ovarian insufficiency and fertility preservation.

Unlike previous studies demonstrating an increased lifespan with rapamycin treatment^13^ we did not observe these effects in our KO or WT mice. However, this may be due to a difference in the timing of the rapamycin diet as we started in our mice immediately after weaning at 3 weeks of age compared to 270–600 days of age. Although we also did not note any significant increase in morbidity or mortality among the rapamycin-treated mice, it is certainly possible that early and long-term use of rapamycin may have fertility-unrelated adverse effects, or annul potential beneficial effects.

The primary limitation of this study was the limited number of mice used. While the experiments included 84 female mice, and utilized many more males and females for breeding and maintenance, the number per histologic time-point (n = 8) and per breeding group (n = 10) limited our power to detect differences in the genotypes. A larger study with more frequent breeding cycles between 37–53 weeks of age will be needed to make strong conclusions on the differences in reproductive lifespan and effects of rapamycin treatment.

Our reproductive and histologic findings are consistent with our hypothesis that the complete loss of functional FMR1 protein and change in mTOR signaling, results in the premature recruitment and depletion of the primordial follicle pool in a novel Fmr1 KO mouse model. Further investigation is needed to elucidate the role of Fmr1 on oocyte development and the potential therapeutic applications for Mtor inhibitors on oocyte development and conditions associated with diminished ovarian reserve.

## Conclusions

Fmr1 KO mice exhibited an earlier decline in fertility, increased follicular activation and elevated mTOR activity. Reversal of phenotypes and protein levels in rapamycin-treated KO mice, as well as increased reproductive lifespan of rapamycin-treated WT mice, suggest the mTOR pathway as a potential therapeutic target.
